# Deep Learning Techniques to Improve the Performance of Olive Oil Classification

**DOI:** 10.3389/fchem.2019.00929

**Published:** 2020-01-17

**Authors:** Belén Vega-Márquez, Isabel Nepomuceno-Chamorro, Natividad Jurado-Campos, Cristina Rubio-Escudero

**Affiliations:** ^1^Department of Computer Languages and Systems, University of Sevilla, Sevilla, Spain; ^2^Department of Analytical Chemistry, Institute of Fine Chemistry and Nanochemistry, International Agrifood Campus of Excellence (ceiA3), University of Córdoba, Córdoba, Spain

**Keywords:** olive oil classification, chemometric approaches, GC-IMS method, machine learning, deep learning, feed-forward neural network

## Abstract

The olive oil assessment involves the use of a standardized sensory analysis according to the “panel test” method. However, there is an important interest to design novel strategies based on the use of Gas Chromatography (GC) coupled to mass spectrometry (MS), or ion mobility spectrometry (IMS) together with a chemometric data treatment for olive oil classification. It is an essential task in an attempt to get the most robust model over time and, both to avoid fraud in the price and to know whether it is suitable for consumption or not. The aim of this paper is to combine chemical techniques and Deep Learning approaches to automatically classify olive oil samples from two different harvests in their three corresponding classes: extra virgin olive oil (EVOO), virgin olive oil (VOO), and lampante olive oil (LOO). Our Deep Learning model is built with 701 samples, which were obtained from two olive oil campaigns (2014–2015 and 2015–2016). The data from the two harvests are built from the selection of specific olive oil markers from the whole spectral fingerprint obtained with GC-IMS method. In order to obtain the best results we have configured the parameters of our model according to the nature of the data. The results obtained show that a deep learning approach applied to data obtained from chemical instrumental techniques is a good method when classifying oil samples in their corresponding categories, with higher success rates than those obtained in previous works.

## 1. Introduction

Olive oil is a fatty substance which is obtained from the fruit of the olive tree *Olea europea L*.. There are three different olive oil categories that in descending order of quality are named as extra virgin olive oil (EVOO), virgin olive oil (VOO), and *lampante* olive oil (LOO). The first two are edible while the last one should be refined prior to be consumed. The EVOO flavor is characterized by a pleasant balanced flavor of green and fruity sensory characteristics. In the VOO and LOO, some negative attributes (chemical compounds associated to defects) can be detected in different proportions. The EVOO is the only non-defective olive oil and therefore it is the most appreciated and expensive. Moreover, selling lower quality olive oils as EVOO is one of the most common olive oil commercial frauds. The classification of olive oil depends on (i) chemical parameters such as free acidity, peroxide value and absorbance (K270 and K232) defined by the current European Union Regulation (EEC, 1991) and (ii) a sensory assessment by trained tasters. The sensory assessment methodology is slow and expensive. Consequently, instrumental analytical measurements used in conjunction with chemometric methodologies represent an alternative for reducing costs in the task of differentiating between olive oil categories.

Few studies (Borràs et al., 2015; Borràs et al., 2016; Garrido-Delgado et al., 2015; Sales et al., 2017; Contreras et al., 2019b) can be found to demonstrate the potential of analytical instruments in order to complement the sensorial analysis to classify olive oil samples as EVOO, VOO, and LOO. To demonstrate the usefulness of these methods, the amount of analyzed samples of different harvests should be high in order to obtain representative conclusions. Also, the accuracy of the classification models could be assessed by splitting the total number of analyzed samples in training and testing sets. And finally, the selection of the correct chemometric approaches would be a key point to offer a method which could classify olive oil with guarantee.

Machine learning algorithms have been used in chemistry for several decades obtaining successful results (Svetnik et al., 2003; Du et al., 2008). The massive use of these algorithms has been due to the fact that they create intuitive models which transform complex input chemical data to an explainable output. However, in more sophisticated chemical problems, the relationships between input data and output solutions are not so easy to identify. Apart from that, some machine learning algorithms are not efficient enough in dealing with high-dimensional data when no dimension reduction is performed. Neural networks solve most of the problems that arise with the use of machine learning algorithms: firstly, they solve the problem of searching and identifying existing relationships, resulting black-box models that are not so interpretable, but with a high level of accuracy. Lastly, there is no problem with the amount of data, that's why they can work efficiently with high-dimensional data.

The use of artificial intelligence to detect the quality of gastronomic and agricultural products is not a new research field. In particular, Deep Learning techniques are being used for similar classification tasks with promising results, for example in the detection of different types of wine using taste sensors and neural networks (Riul et al., 2004) and in food classification (Dȩbska and Guzowska-Świder, 2011). There are also several works with the objective of determining the quality of olive oil with artificial neural networks, as expressed in the review from Gonzalez-Fernandez et al. (2019), however, none of them distinguishes among the three currently existing categories (EVOO, VOO, and LOO), they only distinguish between two (EVOO/non EVOO, LOO/non LOO).

Our aim in this study has been the application of Deep Learning techniques to a group of significant markers obtained by analytical instrumentation, specifically based on gas chromatography coupled to ion mobility spectrometry (GC-IMS). This approach has been applied to 701 samples of the categories EVOO, VOO, and LOO, from two different olive oil harvests (2014–2015 and 2015–2016). The study has been divided in two parts: on the one hand we have studied the two crops covering the years 2014–2016 with the aim of improving the results obtained in a work related to the same dataset (Contreras et al., 2019b) and on the other hand we have applied well known algorithms in the literature to these same harvests in order to compare them with our methodology.

The article is organized as follows: section 2 provides a detailed description about the technique used to obtain the data and the algorithm and methodologies applied to carry out the classification task. Section 3 shows the results obtained with the previous techniques, and finally, section 4 samples the conclusions that have been obtained after the study.

## 2. Materials and Methods

**Figure 1:**
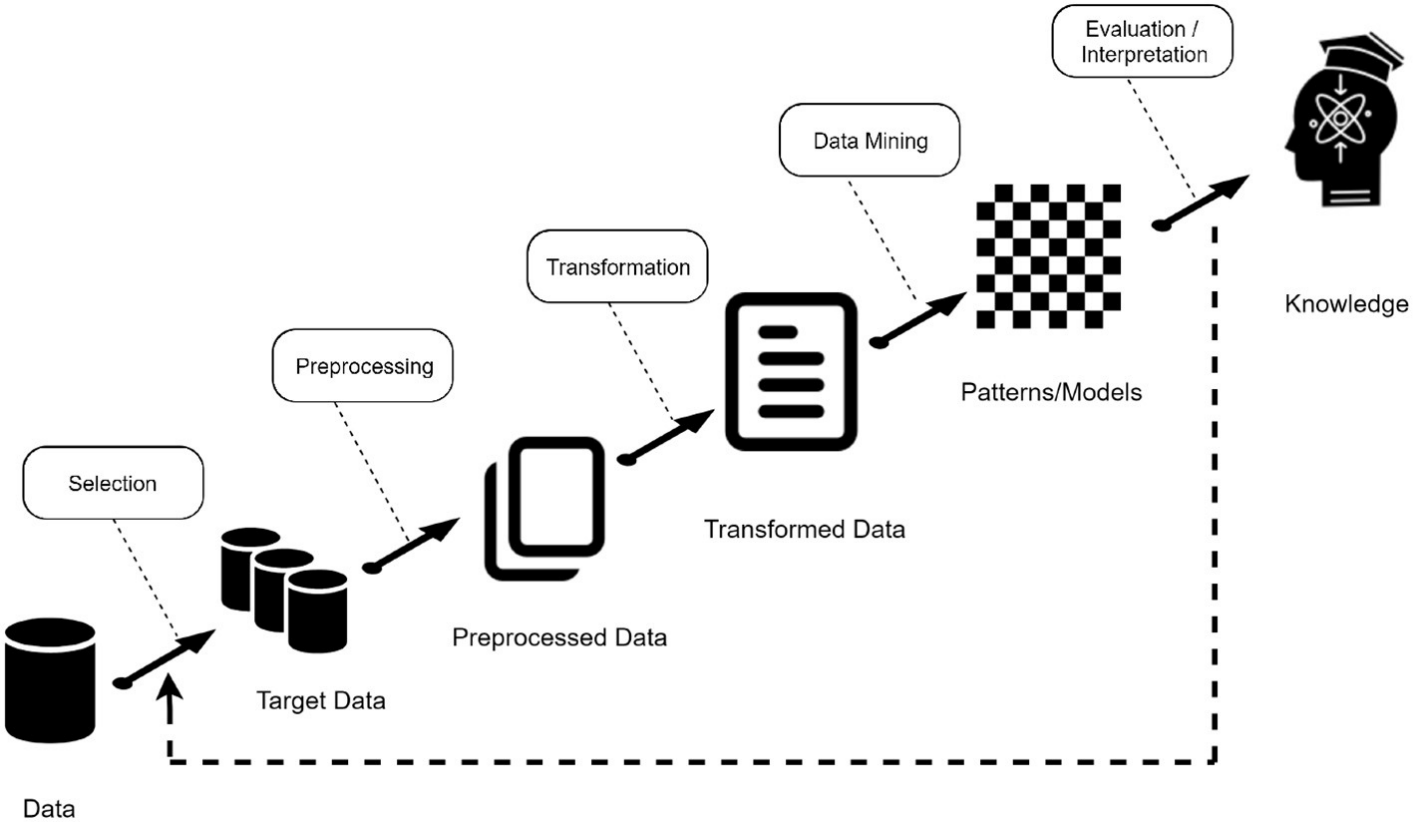
Steps in the data analysis methodology.

In this study, we aimed at providing a data mining approach based on Deep Learning techniques to classify olive oil samples based on chemical data. The main goal is to provide a computational methodology to help and complement the standardized sensory analysis according to the panel test method (Circi et al., 2017). The process followed is known as Knowledge Discovery in Databases (KDD). According to Lara Torralbo (2014), the KDD process pursues the automated extraction of non-trivial, implicit, previously unknown and potentially useful knowledge from large volumes of data. In summary, it can be said that KDD is a term that refers to the whole process of knowledge extraction encompassing certain phases or stages as can be seen in Figure 1.

The stages can be summarized as follows:

- Data acquisition and selection: In this phase, data from different sources are integrated into a single data repository, creating a target dataset with interesting variables or data samples, on which discovery is to be performed.
- Preprocessing: It might not be possible to perform data mining on the data collected in the dataset, because the data may not be clean, may contain irrelevant attributes, etc. Different types of data selection, cleaning and transformation techniques are applied in this phase, e.g., feature selection, data cleaning.
- Transformation: The data mining algorithms that will be used in the later phase sometimes need to have a specific data input format. The transformation phase is in charge of this task, with techniques such as normalization or auto-scaling.
- Data Mining: this part of the process is in charge of solving the main problem presented, using classification, regression, among others.
- Evaluation: After obtaining the data mining models, the last step of the KDD process consists of evaluating the quality of these models and interpreting them to obtain the desired knowledge. In general, in order to evaluate a model, a small subset of the data (test set) is reserved and used to validate the model built with the rest of the data (training set). This approach is known as simple validation.

This process is not static, that is, it can vary depending on the problem, taking into account the nature of the data chosen to decide whether to follow all phases, add extra phases or just follow some of them. We have mainly carried out four stages: data acquisition, data visualization techniques, data preprocessing, classification models and finally a validation stage of the proposed model.

### 2.1. Data Acquisition

#### 2.1.1. GC-IMS Analysis

Analyses of olive oil samples were carried out with a GC-IMS commercial instrument (FlavourSpec^Ⓡ^). The IMS module was equipped with a tritium radioactive ionization source of 6.5 KeV and a drift tube of 5 cm long (Gesellschaft für Analytische Sensorsysteme mbH, G.A.S., Dortmund, Germany). A non-polar column (94% methyl-5% phenyl-1% vinylsilicone) with 30 m of length, an internal diameter of 0.32 mm and 0.25 μm of film thickness (SE-54-CB of CS-Chromatographie Service GmbH, Düren, Germany) was coupled to the IMS device. In addition, an automatic sampler unit (CTC-PAL, CTC Analytics AG, Zwingen, Switzerland) was employed to improve the reproducibility of measurements. The GC-IMS method for olive oil analysis was obtained from a previous work by Contreras et al. (2019b). The sample introduction system employed was a headspace generated in a 20 mL glass vial closed with magnetic cap and silicone septum. Then, 1 g of olive oil was placed in that vial and the sample was heated at 60°C for 8 min. The automatic injection of 200 μL of headspace was carried out with a heated syringe (80°C) into the heated injector (80°C). The injected headspace was driven into the GC column by using nitrogen 5.0 as carrier gas at 5 mL min^−1^ the first 6 min and then it was increased to 25 mL min^−1^ until the end of the analysis (23 min). Neutral analytes were separated at 40°C. Later, this neutral volatiles were introduced into the IMS ionization chamber to generate their corresponding ions. The generation of ions of this IMS device takes place due to the presence of an excess reagent whose signal is called reactant ion peak (RIP) which is always registered in the measurements. In positive polarity, the RIP consist on hydrated protons generated due to the collision of primary electrons emitted by the tritium source with nitrogen, and a subsequent series of reactions. When one analyte (M) enters into the ionization chamber, the corresponding ion is formed due to the association of M to this hydrated proton resulting in the displacement of water molecules (Jurado-Campos et al., 2018). Then, the ions were separated in the drift tube working at a constant temperature and voltage of 55°C and 400 V cm^−1^, respectively. A counter-current gas flow of nitrogen was also used (drift gas) at a 250 mL min^−1^ rate. This flow is necessary to eliminate neutral molecules in the drift tube and influences the separation of ions in it. The values of different IMS parameters were set at: 32 for average of scans for each spectrum acquired, 100 μs for grid pulse width, 21 ms for repetition rate and 150 kHz for sampling frequency. Finally, two-dimensional GC-IMS data were acquired in positive mode, represented as topographic plots in LAV software (version 2.0.0) from G.A.S. So that, each individual signal or marker included in these 2D maps is characterized by the retention time of the neutral compound in the GC column, the drift time of the ion generated in the IMS (the time that the swarm of ions spend traveling along the drift tube) and its intensity value which depends on the concentration. The intensity of each marker can be automatically obtained from the topographic plots using LAV quantification module tool of the software.

#### 2.1.2. Datasets

We analyzed 292 olive oil samples from the 2014–2015 harvest and 409 samples from the 2015-2016 harvest, henceforth named datasets D1, and D2. For D1, the 292 olive oil samples are divided in 98 EVOO, 159 VOO and 35 LOO samples. D2 harvest was composed by 92 EVOO examples, 196 VOO and 121 LOO.

The structure of the dataset for harvest D1 and D2 is the same, i.e., the datasets have a total of 118 attributes, with 113 being intensity of the markers (Contreras et al., 2019b) and the remaining others indicate the identifier of the sample (“Name”), the class (EVOO, LOO, VOO) to which it belongs (“Class”), the base value (“Baseline”), the position of the RIP (“RIP Position”) and the maximum intensity of the RIP (“RIP Height”) respectively.

### 2.2. Visualization

Before applying data analysis techniques it is important to know the nature of the data. The stage of visualization undertakes this task. In this section we provide some graphical information about the dataset analyzed. In particular, two different visualizations have been carried out: first, we show the proportion of each type of olive oil sample using pie charts and second, we reported results from principal component analysis to describe possible partitions in the dataset.

**Figure 2:**
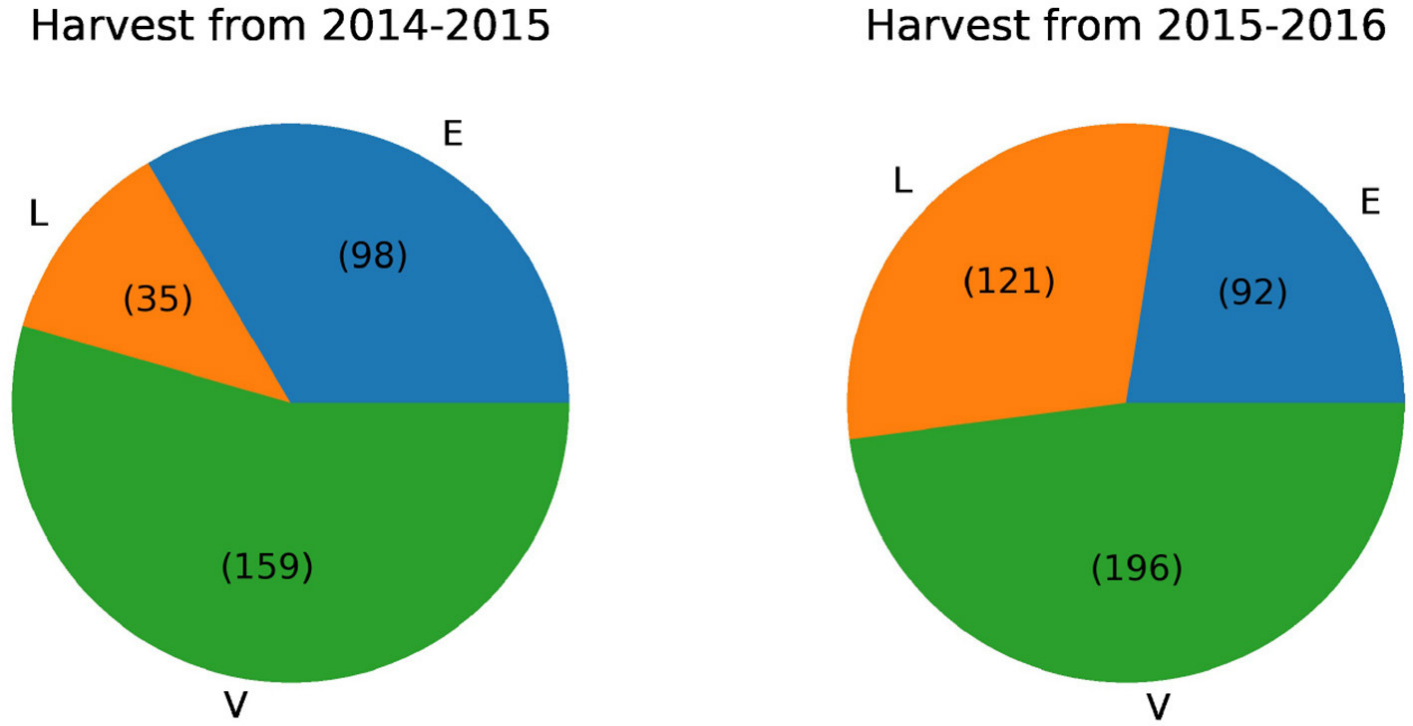
Number of instances for each olive oil class in harvests from 2014 to 2016.

Figure 2 reports the proportion of each type of olive oil in the different harvests using a pie plot graphic. It can be seen that the two harvests have very few instances of EVOO compared to the last. For this reason we decided to merge these harvests into one. This union serves to improve the classification algorithm results since the training set will have more instances. After this union the distribution of instances is 190 EVOO, 355 VOO, and 156 LOO.

**Figure 3:**
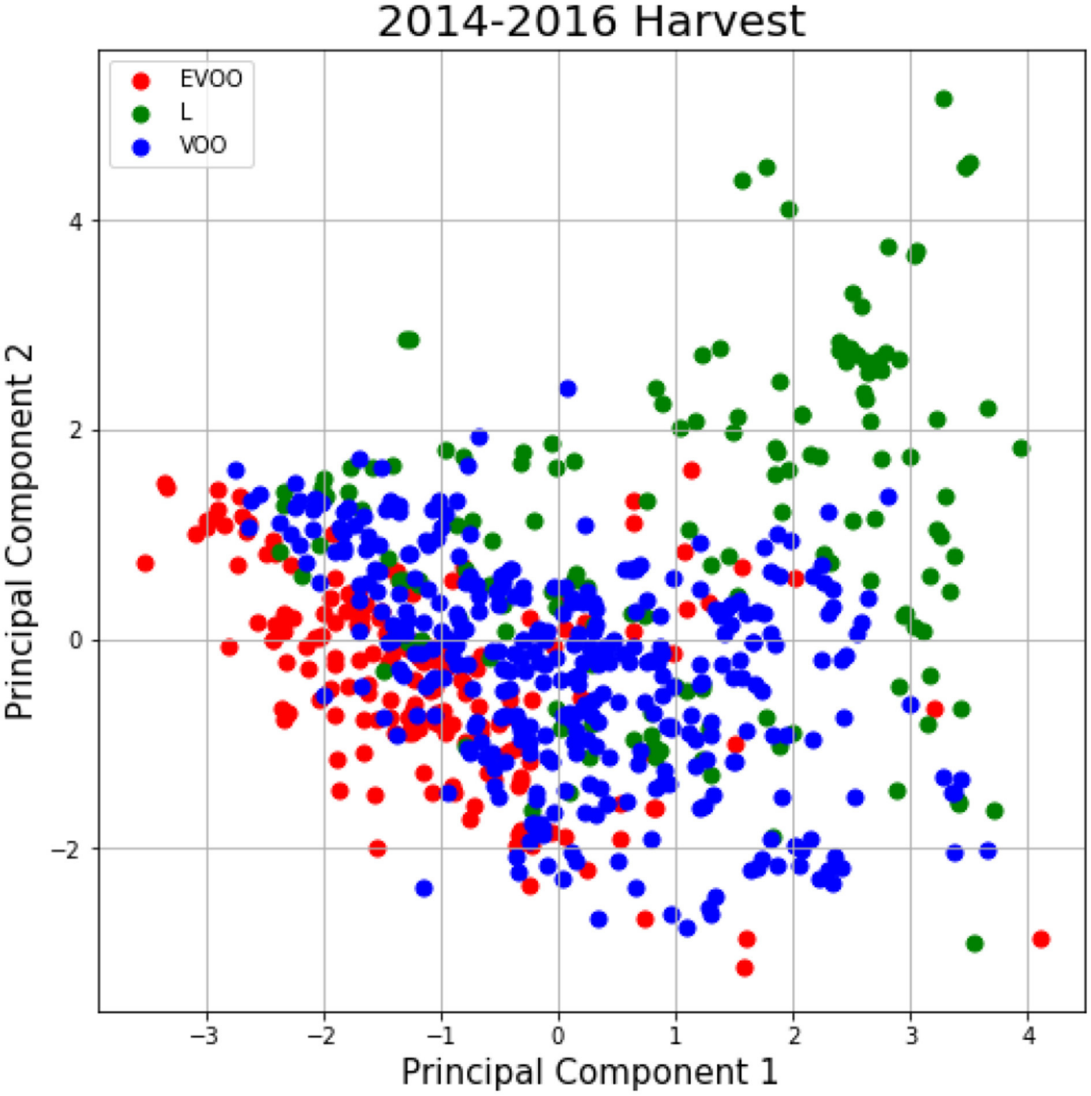
PCA for the 2014–2016 harvest.

Furthermore, a principal component analysis (PCA) has been carried out. This study aims at a priori determination of the number of possible existing partitions. Figure 3 illustrates data distribution into the first two components of PCA-analysis for 2014–2016 harvest. According to these figures there is not an a priori clear separation among classes, and therefore we decide to apply Deep Learning techniques to this problem. Deep learning techniques are able to learn a meaningful latent space, i.e., find and represent relationships among attributes that are not known a priori and are suitable for the olive oil classification problem.

#### 2.2.1. Preprocessing

Two fundamental tasks were carried out in the preprocessing phase: the normalization of samples with respect to RIP Height in order to reduce potential instrumental variations and auto-scaling of markers that may improve the results obtained in the classification task. First, the normalization is made by dividing each of the values of markers for the maximum value of the RIP, in order to work with more homogeneous data. Second, after carrying out several tests, we found out that the auto-scaling (sometimes also called, standardization, or z-transformation) of markers resulted in slightly improved classification results. Thus, each column of the dataset was auto-scaled, i.e., numeric columns will have zero mean and unit variance. The equation used to do this task is the following:

(1) $$\begin{array}{r} z_{1}=\frac{x_{i}-\bar{x}}{s} \end{array}$$

where:

- *z*_1_: marker auto-scaled,
- *x*_1_: marker we want to auto-scale,
- $\bar{x}$: mean of the values for the marker,
- *s*: sample standard deviation.

#### 2.2.2. Classification Task

**Figure 4:**
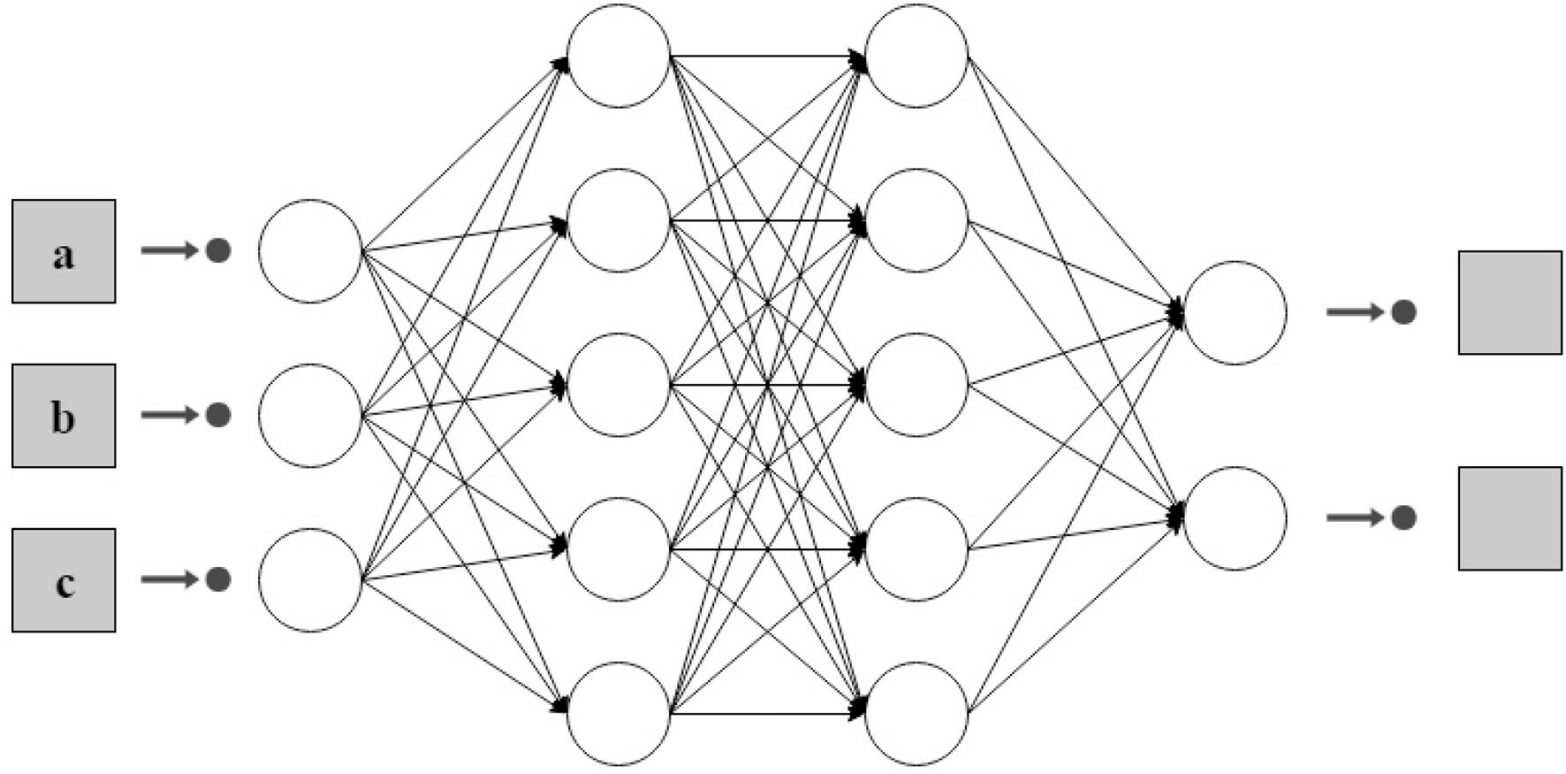
An example of and architecture of two hidden layers for a dataset with three attributes and two possible classes.

For the classification task, a feed forward artificial neural network was used. An artificial neural network is a computational learning algorithm based on the architecture of the biological neural networks of the brain (Gibson and Patterson, 2016). These networks seek at finding a function that approximates data input into a desired output (DeepAI contributors, 2018). The architecture of an artificial neural network is determined by three main elements, nodes, connections between nodes and layers. Nodes are elements that try to model the neurons of the biological brains. The connections between nodes, such as synapses in brains, allow signals to be transmitted from one node to another. The combination of neurons are called a layer, the set of one or more layers constitutes the neural network. There are three types of layers: input, hidden and output. The input layer is composed of neurons that receive data of the problem that is under study. In this case, the input layer obtains the data of each of the features of the dataset, in our problem markers of the harvests. The hidden layers are those between the input and the output, so they do not have a direct connection to the environment. The output layer is the one that is responsible for providing the classification result obtained after applying the learning algorithm. Depending on the number of layers and the direction in which the information flows, several types of neural networks can be distinguished (Larranaga et al., 2019). A multilayer feed forward network (Gibson and Patterson, 2016) has been used to classify olive oils in our study. A multilayer feed-forward network is a neural network with an input layer, one or more hidden layers, and an output layer where each layer has one or more artificial neurons as can be seen in Figure 4.

**Input layer**. This is the first layer of a feed forward neural network. It receives the information of the problem, i.e., the input dataset. The number of neurons in this layer is usually the same as the number of attributes of the problem under study. Input layers in classical feed-forward neural networks are fully connected to the next hidden layer.

**Hidden layer**. The number of hidden layer in a feed forward neural network depends on the problem. Hidden layers are in charge of encoding and transporting the information extracted from the dataset to the following layers. These layers are also the key that allow neural networks to model non-linear functions.

**Output layer**. This layer is the one that allows to obtain the prediction of the model on the data. Depending on the nature of the problem, this prediction can be a real value (regression) or a set of probabilities (classification). To obtain these values, the corresponding activation function is chosen. In our case we have chosen the *softmax* function that represents the distribution of probability over *K* different outputs. In our example, the output is a vector with three values (or two values depending on whether the model is ternary or binary) that indicates the probability that an example belongs to one class or another.

#### 2.2.3. Validation

The previous study carried out on this same dataset (Contreras et al., 2019b) used the accuracy as the validation metric. In order to compare with the previous results we decided to take this measure to validate the generated model. Accuracy is defined as the percentage of correctly classified examples from the dataset. To calculate it, it is necessary to take a look at the confusion matrix. If we define two variables, P for the positive instances and N for the negative ones, a confusion matrix is a table that allows for the visualization of the performance of an algorithm, typically a supervised learning one. It is a table with four different combination values: the rows indicate the predicted values by model and the columns represent the actual value of the class.

Taking into account the values of the confusion matrix, the accuracy score can be defined as follows:

(2) $$\begin{array}{r} accuracy=\frac{TP+TN}{TP+FP+FN+TN} \end{array}$$

where:

- TP (True Positive): values correctly classified as positive.
- FP (False Positive): Predicted values with negative label but which actually belong to the positive class.
- FN (False Negative): incorrectly predicted as negative values because their real value is positive.
- TN (True Negative): correctly predicted values as negative since they actually belong to the negative class.

In multi-class classification with N classes, the confusion matrix has N*N different values and the accuracy score can be obtained in two different ways by the *one vs. all* approach or by the *one vs. one*. The one vs. all approach involves training a single classifier per class, with the samples of that class as positive samples and the remaining as negatives. Finally, accuracy is obtained as a mean of each of the accuracy obtained individually for each class. In the other hand, the one vs. one approach considers each binary pair of classes and trains the classifier on a subset of data containing those classes. During the classification task, each classifier predicts one class, and the class which has been predicted the most is the answer (voting scheme). In this case, one vs. all methodology was used.

Due to the imbalance between the classes, we have also decided to take into account other more appropriate measures: sensitivity and specificity. This measures can be defined as follows:

(3) $$\begin{array}{r} sensitivity=\frac{TP}{TP+FN} \end{array}$$

(4) $$\begin{array}{r} specificity=\frac{TN}{FP+TN} \end{array}$$

### 2.3. Software and Experimental Setting

The neural networks used in this study have been implemented with the Keras library (Chollet et al., 2015). Keras is a high-level neural networks API (application programming interface), written in Python and capable of running on top of Tensorflow. The standardization of the data as well as the division of the training set in train and test has been carried out with the scikit-learn library (Pedregosa et al., 2011). The selection of parameters of the model for each of the harvests involved executing the code as many times as the number of possible neurons in the hidden layer. Due to the large amount of data available, the executions were performed on an Intel machine, specifically Intel(R) Core(TM) i7-8700 CPU @ 3.20 GHz, with 64 GB of RAM and 12 cores. The source code with the different tests performed in this study can be found in Vega (2019).

## 3. Results

### 3.1. Preprocessing of the Data

First, a preprocessing step was performed, this step includes two sub-processes: in the first place a normalization of the data with respect to the maximum height of the RIP was carried out, i.e., each one of the samples is divided by the maximum value of intensity found in each one of them, in order to avoid the variations that can be introduced by the instrumental equipment used. Second, an auto-scaling of the data was carried out since as a previous study (Han et al., 2003) showed that data auto-scaling is a necessary step to improve final classification results. Furthermore, LeCun et al. (2012) have shown that the convergence of Deep Learning models is usually greater if the mean of each of the variables of the training set is close to zero. Because of this, we have auto-scaled the data in order to obtain better results with Deep Learning techniques.

**Figure 5:**
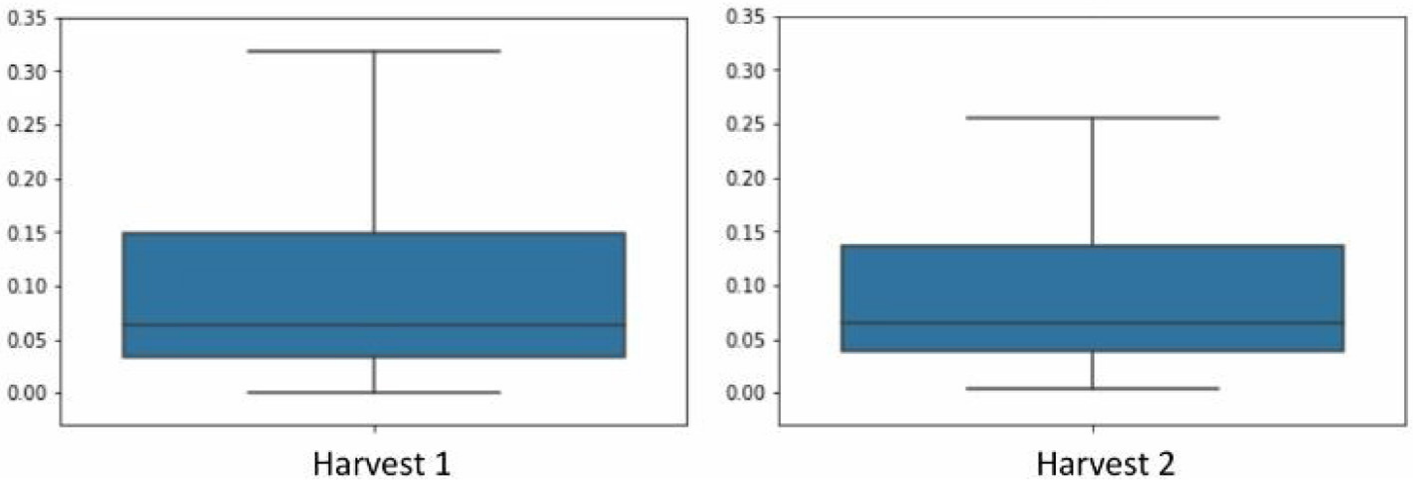
Distribution of averages of each marker for each of the harvests.

As we mentioned before, the chemical method used to obtain the data from D1 and D2 are the same being the number of markers equal for each case. Thus, after data auto-scaling, a union of the datasets D1 and D2 was carried out in order to study them as a whole, henceforth named D1–D2. We could observe in the Figure 5 that the distribution of averages for each column of the dataset were very similar between D1 and D2, which is another motivation behind our decision to merge the two crops.

### 3.2. Use of Deep Learning Models for Classification of Olive Oil Samples

The capabilities of a neural network to make good predictions depends on its architecture and its parameters, it is an essential task to define a well structured network before implementing the model. Parameters which define the model architecture are known as hyperparameters and the process of assessing the best configuration for those parameters is called hyperparameter tuning (Diaz et al., 2017).

For the present study, multilayer and unidirectional (feed-forward) neural networks have been used, with an input layer, a hidden and an output layer, with a flow of information that run from the entrance to the exit, only in one direction.

The first step was to improve the classification of the model varying the values for the activation function and optimization algorithm. The best results were obtained with Rectified Linear Unit function (RELU) and Adam algorithm, respectively. The second step was to choose the optimal number of hidden layers for this particular problem. Finally, the number of the neurons in the hidden layers was optimized. Taking as a guide the rules of thumb (Heaton, 2008) and the geometric pyramid rule (Masters, 1993) that will be explained below, experiments were performed for datasets D1–D2 as a whole. In each of these experiments tests were made varying the number of neurons looking for the number that provided the best results.

#### 3.2.1. Choosing the Number of Hidden Layers

The universal approximation theorem (Csáji, 2001) states that a feed-forward network with only a single hidden layer containing a finite number of neurons can approximate continuous functions as well as other interesting functions when appropriate parameters are given. The use of more than one hidden layers are better for complex datasets than involves time-series or computer vision. The dataset of this study does not belongs to any of these two categories, so we considered that one hidden layer is the best approach.

#### 3.2.2. Choosing the Number of Neurons in the Hidden Layer

Deciding the correct number of hidden layers is only one part of the problem. The correctness of the model also depends on the number of the neurons in the hidden layers. There are a lot of theorems that provide a first approximation for this issue. The one selected for our research is called “geometric pyramid rule” proposed by Masters in Masters (1993). Basically, this rule asserts that there is no magic formula for selecting the optimum number of hidden neurons although it provides a rough approximation for different structure, e.g., for a three layer network with n input and m output neurons, the hidden layer would have $\sqrt{n\times m}$ neurons.

Besides the geometric pyramid rule, a few rules of thumb methods (Heaton, 2008) have been considered for determining an acceptable number like the following:

- The number of hidden neurons should be between the size of the input layer and the size of the output layer.
- The number of hidden neurons should be 2/3 the size of the input layer, plus the size of the output layer.
- The number of hidden neurons should be less than twice the size of the input layer.

Taking into account these previous rules, we decided to train the model for each of the possible combinations of neurons considering the inputs and outputs of the neural network according to the harvests under study: for D1-D2, tests were carried out varying the neurons from 2 to 3 to 113 depending on whether the model distinguishes between two classes (binary model), that is, between lampante and no lampante (LOO/non-LOO) or extra or no extra (EVOO/non-EVOO), or between three (EVOO/VOO/LOO). The Table 1 shows among all the possible values of neurons, the one that maximizes the accuracy value for each of the tests. As it can be seen in this table, the number of neurons for each case is completely different, there is no single number that ensures the total quality of the model. Although this process has been very time consuming, it is totally necessary since it is the first time that Deep Learning techniques have been used with these specific data, so it was convenient to see each one of the cases. For future studies we propose training the neural network with many more examples in order to further homogenize the parameter selection of the model.

**Table 1:** Number of neurons chosen for the hidden layer.

	**2014–2016 (D1-D2)**	
	**Non-standardized**	**Standardized**
EVOO/VOO/LOO	32	40
EVOO/non-EVOO	10	3
LOO/non-LOO	68	53

### 3.3. Training the Model

A train-test split method was used for the validation of the model. A training set containing 80% of the samples was used for the calibration of the models and the remaining 20% of the samples were used as a validation or blind test. The performance of the neural network was shown by the accuracy score.

A total of 6 tests with the data from D1 to D2 has been carried out. The models were tested with auto-scaled and non auto-scaled data, as well as the division of tests according to the type of oil. For each of the tests the optimum number of neurons in the hidden layer has been calculated, so that for each test a model has been made for each of the possible neurons in the hidden layer according to the rules described above, specifically 110 iterations for the model that discriminates between the 3 classes (the number of neurons must be between the output number and the number of input neurons) and 111 for those that distinguish between two classes.

#### 3.3.1. Results Obtained for 2014–2016 Harvests

A total of 701 samples from 2014–2016 harvests were studied. The Deep Learning model was built using 80% of these samples (a total of 531 olive oil samples, of which 286 where VOO, 149 EVOO, and 126 LOO) and the remaining 20% to evaluate the model (69 VOO, 41 EVOO, and 30 LOO). To compare our results to those obtained by Contreras et al. (2019b), we have replicated each of their tests, obtaining 3 different models: 2 binary models and 1 ternary model. The first binary model allows to differentiate between EVOO and non-EVOO examples, the second model discriminates between LOO and non LOO, and finally, the ternary model discriminates between all classes, i.e., among EVOO, LOO, and VOO. As mentioned above, auto-scaling seemed to be a good preprocessing task that should be carried out with this dataset, so the three models obtained have also been carried out in two different ways, first without auto-scaling the data and second with auto-scaled data.

Table 2 shows the comparison of results between our study and the existing previous study as well as the accuracy increase ratio. We can observe that our results improve the results obtained without auto-scaling (see column 2 and 3). Furthermore, the part in which our results are shown verifies that a previous preprocessing of the data is a good technique, as it improves the results in comparison to those obtained without this preprocessing. On the other hand, if we compare our results with the previous results, we see a significant improvement for each of the three models studied, with the rate of increase always positive.

**Table 2:** Results obtained for 2014–2016 harvests.

	**Previous results**	**Our Results**		**Rate of increase (%)**
		**Non standardized**	**Standardized**	
EVOO/VOO/LOO	74.29	80.71	81.42	9.59
EVOO/non-EVOO	85.72	88.57	90.00	4.99
LOO/non-LOO	90.71	94.28	95.00	4.72

### 3.4. Comparison to Other Methods

Our methodology has been compared to five different benchmark methods: K-Nearest Neighbors (Altman, 1992), Support Vector Machine (Boser et al., 1992), Decision Tree Classifier (Safavian and Landgrebe, 1991), Logistic Regression (Scott et al., 1991) and XGBoost (Chen and Guestrin, 2016). The data used for comparison are the auto-scaled data, since the objective of this comparison is to provide a comparative framework on the best results obtained with the proposed methodology.

We have evaluated these methods for D1-D2 harvest data. Firstly, for accuracy score (Table 3), in EVOO/VOO/LOO model, among the five models used for comparison, XGBoost offers the best performance. In the case of EVOO/non-EVOO model, XGBoost is as good as k-NN. Lastly, LOO/non-LOO model gets higher performance with Logistic Regression. Although the results are quite satisfactory with benchmark algorithms, none of the models achieves better results than our Deep Learning proposal if we take into account the average of the three models (last row). It can be seen that the best value (in bold) is always the one in the first column, which is the one corresponding to Deep Learning. Lastly, for sensitivity (Table 4) and specificity (Table 5) our proposal is the best if we also take into account the average of the three models.

**Table 3:** Accuracy comparison with other methods for 2014–2016 (D1-D2) harvests.

	**Deep learning**	**SVM**	**k-NN**	**Tree**	**Regressor**	**XGBoost**
EVOO/VOO/LOO	81.42	73.57	77.14	68.57	77.85	80.71
EVOO/non-EVOO	90.00	85.71	85.71	82.14	85.71	86.42
LOO/non-LOO	95.00	90.00	90.71	84.28	92.85	90.00
	**88.81**	83.09	84.52	78.33	85.47	85.71

**Table 4:** Sensitivity comparison with other methods for 2014–2016 (D1-D2) harvests.

	**Deep learning**	**SVM**	**k-NN**	**Tree**	**Regressor**	**XGBoost**
EVOO/VOO/LOO	63.47	55.82	59.33	49.76	61.52	64.11
EVOO/non-EVOO	68.29	68.29	63.41	60.97	68.29	68.29
LOO/non-LOO	80.00	56.66	63.33	60.00	76.66	63.33
	**70.58**	60.25	62.02	56.91	68.82	65.24

**Table 5:** Specificity comparison with other methods for 2014–2016 (D1-D2) harvests.

	**Deep learning**	**SVM**	**k-NN**	**Tree**	**Regressor**	**XGBoost**
EVOO/VOO/LOO	87.55	83.57	85.45	80.00	86.58	87.81
EVOO/non-EVOO	93.93	92.92	94.94	90.90	92.92	93.93
LOO/non-LOO	98.18	99.09	98.18	90.09	97.27	97.27
	**93.22**	91.86	92.85	86.99	92.25	93.00

## 4. Discussion

Deep Learning techniques are proving to be one of the best tools when performing complex tasks that require expert knowledge (Arel et al., 2010; LeCun et al., 2015). In this study we used Deep Learning techniques to provide an automatic complement to the panel test method. This is an essential task to avoid fraud in the price and to know whether the olive oil is suitable for consumption or not. This work has shown the feasibility of a feed forward artificial neural networks-based model as a classifier to differentiate EVOO, VOO, and LOO oil from GC-IMS spectroscopy data.

The preprocessing step should be highlighted since the auto-scaling of data has been a fundamental part of the study carried out. This step has meant an improvement in the classification algorithms as can be seen in Table 2.

This study also shows that the neural network architecture must be different for each of the potential models. The fact that the number of neurons in the hidden layer is different for each of the models (binary or ternary) is not surprising; indeed, we would even say it is necessary, due the fact that the network must be adapted to the input data.

Until now, the best works on oil classification (Contreras et al., 2019b; Gonzalez-Fernandez et al., 2019) worked in a similar way to our proposal: they first made a chemical treatment to obtain the data, and then applied some mathematical model to carry out the olive oil classification. The main advantage of our approach is that there is a searching for the most suitable parameters, thus achieving a better adaptation to the input data to achieve the most accurate results.

One of the objectives of this work has been trying to improve the results obtained by Contreras et al. (2019a) with D1 and D2 harvests. Considering that in that previous work they obtained an accuracy of the 74.29% using techniques such as PCA and OPLS-DA, our work, with an accuracy of 81.42%, has shown that Deep Learning techniques are a very useful tool to classify olive oil samples from GC-IMS data.

Additionally, regardless of the number of neurons used, the best results are obtained for binary models, especially the model that classify between LOO and non-LOO. This may be due to the fact that in the case of the ternary model, the elements are more difficult to split since the VOO is at the crossroads between EVOO and LOO, which means that the separation between classes is not so clear.

We have also studied the performance obtained by five different benchmark methods: k-Nearest Neighbors, Support Vector Machine, Logistic Regression, Decision Tree Classifier and XGBoost. Although the performance of these algorithms is satisfactory, in none of the cases they have improved our Deep Learning approach.

Finally, some limitations of our study should be noted and discussed. First, it is known that the success of a Deep Learning algorithm lies in the amount of data available to train. In this case, we have only a total of 701 examples. For further studies, we propose to create synthetic data with Conditional Generative Adversarial Networks as proposed in Vega-Márquez et al. (2020). Lastly, another major problem we have encountered is the imbalance between classes, in olive oil industry is common to have more instances from VOO that LOO and EVOO. In order to address this issue we propose to employ Machine Learning algorithms as SMOTE (Chawla et al., 2002) to balance classes.

## Data Availability Statement

The datasets for this manuscript are not publicly available because the data has been obtained and treated only for analysis as shown in this article. For any further use new access has to be granted. Requests to access the datasets should be directed to Cristina Rubio-Escudero, crubioescudero@us.es.

## Author Contributions

BV-M has contributed with the implementation, design of tests, and manuscript redaction. NJ-C has contributed with the data acquisition, first analysis, and manuscript redaction. IN-C and CR-E have contributed with the implementation and tests supervision and manuscript correction.

### Conflict of Interest

The authors declare that the research was conducted in the absence of any commercial or financial relationships that could be construed as a potential conflict of interest.
